# Reassortment compatibility between PB1, PB2, and HA genes of the two influenza B virus lineages in mammalian cells

**DOI:** 10.1038/srep27480

**Published:** 2016-06-08

**Authors:** Jin Il Kim, Ilseob Lee, Sehee Park, Joon-Yong Bae, Kirim Yoo, Philippe Lemey, Mee Sook Park, Jin-Won Song, Sun-Ho Kee, Ki-Joon Song, Man-Seong Park

**Affiliations:** 1 Department of Microbiology, the Institute of Viral Diseases, College of Medicine, Korea University, Seoul 02841, Republic of Korea; 2 Department of Microbiology and Immunology, Rega Institute, KU Leuven – University of Leuven, Leuven 3000, Belgium

## Abstract

In addition to influenza A subtypes, two distinct lineages of influenza B virus also cause seasonal epidemics to humans. Recently, Dudas *et al.* have done evolutionary analyses of reassortment patterns of the virus and suggested genetic lineage relationship between PB1, PB2, and HA genes. Using genetic plasmids and reassortant viruses, we here demonstrate that a homologous lineage PB1-PB2 pair exhibits better compatibility than a heterologous one and that the lineage relationship between PB1 and HA is more important for viral replication than that between PB2 and HA. However, co-adaptation of PB1-PB2-HA genes appears to be affected by complete gene constellation.

In contrast to the diverse host range of influenza A, influenza B virus (IBV) mainly infects humans^1^,^2^. No subtype classification has been applied to this virus, but the two antigenically distinct lineages, so-called Victoria (B/Victoria/2/87) and Yamagata (B/Yamagata/16/88), have diverged since mid 1980s^3^. This has been one of many reasons why selecting an appropriate vaccine antigen against the IBV proved challenging^4^,^5^.

Based on the analysis of the eight genetic segments, Dudas *et al.* reported that only PB1, PB2, and HA genes of IBVs maintain two separate lineages whereas the other segments are frequently exchanged between the Yamagata (PA, NP, NA, and M genes) and Victoria (NS gene) lineages^6^. Also, these three genes appeared to be reassorted together according to their genetic lineages. This suggests co-adaptation between PB1, PB2, and HA genes and selection against reassortants that result from lineage mixing for these genes. Using 431 complete IBV genomes (Data S1), we first reconstructed phylogenetic relationships of the eight genes of IBVs. These appeared to be similar to the results of Dudas *et al.*, except for the NS gene. We observed an independent lineage of recent NS genes instead of the ancestry from either Victoria or Yamagata lineage, which might be caused by the different sets of genomic sequences used in both studies. However, our study also suggests the same Czechoslovakia strain (B/Czechoslovakia/69/1990) as the representative of recent NS genes (Fig. 1 and Fig. S1).

For the PB1-PB2-HA co-adaptation, our reassortment analysis also detected their homologous relationship since 1998 whereas some minor cases of heterologous lineage sets were only detected between 1992 and 1995 (Table 1). For most putative reassorted viruses, it was revealed that both PB1 and PB2 genes appeared to be donated by one virus whereas the HA might be contributed from the other (Table 1). These again suggest selection against the viruses harboring mixed lineage sets of PB1, PB2, and HA genes, and this evolutionary force might eventually affect overall gene constellation of IBVs.

To address the virological significance of these evolutionary reconstructions, we investigated reassortment compatibility between PB1 and PB2 genes using a plasmid-based, mini-replicon assay in 293T cells. Even though relative capacity was likely to be different between wild-type B/Brisbane/60/2008 (Victoria lineage, Bris-Vc) and B/Wisconsin/01/2011 (Yamagata lineage, Wisc-Ym) ribonucleoprotein (RNP) complexes (PB1, PB2, PA, and NP), the RNPs having a heterologous PB1-PB2 pair (pB/W:PB1, pB/W:PB2, pW/B:PB1, and pW/B:PB2) exhibited reduced polymerase activity than the wild-type RNPs (pBris-Vc and pWisc-Ym) harboring a homologous pair, and the reduction was larger in the PB1 reassortants (pB/W:PB1 and pW/B:PB1) than in the PB2 ones (pB/W:PB2 and pW/B:PB2) (Fig. 2A).

The reduction in polymerase activity correlated with a decrease in viral replication in Madin-Darby canine kidney (MDCK) cells, but only for the viruses with a Bris-Vc backbone (Fig. 2B–D). The replication titers indicate that compared to the replication capacity of the wild-type Bris-Vc virus (rBris-Vc), either PB1 or PB2 of the Wisc-Ym virus reduced that of the Bris-Vc virus, and the Wisc-Ym PB1 (rB/W:PB1) conferred a much enlarged decrease than the Wisc-Ym PB2 (rB/W:PB2) (Fig. 2B,C). This replication decrease was still observed when both Wisc-Ym PB1 and PB2 genes were combined together (rB/W:PB1,PB2) (Fig. 2D). However, the Wisconsin HA appeared to compensate for the PB1-mediated replication defect by increasing the replication capacity of the rB/W:PB1,HA and the rB/W:PB1,PB2,HA viruses up to the similar replication level of the Wisc-Ym PB2-reassorted viruses (rB/W:PB2 and rB/W:PB2,HA) (Fig. 2B–D). These may indirectly suggest a closer relationship between PB1 and HA than between PB2 and HA, as observed in previous influenza A pandemics in 1957 and 1968^7^,^8^.

Unlike the viruses with a Bris-Vc backbone, the viruses with a Wisc-Ym backbone appeared to be capable of combining the Bris-Vc PB1, PB2, and/or HA genes (Fig. 2E–G). Replication titer changes between the wild-type Wisc-Ym and reassortant viruses were limited, and even, enhanced replication titers were observed with the rW/B:PB1 and rW/B:PB1,HA viruses until 24 hours post-infection (hpi) (Fig. 2F). Given the evolutionary reconstruction reported by Dudas *et al.*, these might indicate why the two lineages of PB1, PB2, and HA genes of IBVs are recombined on the Yamagata genetic background. However, considering that the rW/B:PB1,PB2,HA virus was not generated from our repeated rescue trials, something beyond lineage relationship between PB1, PB2, and HA genes, such as a HA-NA functional balance^9^,^10^,^11^ and undefined interlineage relationship between the Victoria and the Yamagata genes^12^, is also of importance for overall fitness of IBVs. As seen in Fig. S2, genetic divergence of the PA, NA, M, and NS genes of the two viruses used in this study might also affect the results. However, reassortment compatibility between PB1, PB2, and HA genes was all assessed on either Bris-Vc or Wisc-Ym virus backbone, so contribution of the genetic divergence observed in the PA, NA, M, and/or NS genes was not considered in this study.

Based on previously reported analyses and the results from our genomic analysis, we here demonstrate virological evidence of close relationship of the PB1, PB2, and HA genes of IBVs. We suggest that the homologous pair of PB1-PB2 and relationship of PB1-HA may be more important than the heterologous pair of PB1-PB2 and that of PB2-HA in terms of polymerase activity and viral replication in cells, respectively. However, co-adaptation of PB1-PB2-HA set appears to be determined by complete genomic context of IBVs.

## Methods

### Phylogenetic trees

Complete genome sets of 431 IBVs circulating between 1986 and 2012 years were downloaded from the database of the National Center for Biotechnology Information. The eight genetic segment sets of the viruses were aligned using the MAFFT program^13^. Phylogenetic relationships were then reconstructed using a time-framed Bayesian evolution analysis approach via a Markov Chain Monte Carlo (MCMC) inference method, implemented in the BEAST package (v1.8.2)^14^. To reconstruct the evolutionary relationships of each genetic sequence set, we adopted flexible and consistent models for all segments, including a GTR+I+Γ substitution model^15^, a lognormal relaxed molecular clock, and a Bayesian skygrid tree models as a tree prior. MCMC analyses were run for 50 million iterations, sampling every 50 thousand iterations after a 10% burn-in. Two independent runs were ensured for their convergence in Tracer (v1.6) (http://tree.bio.ed.ac.uk/software/tracer/). Maximum clade credibility (MCC) trees were obtained from the MCMC tree samples using TreeAnnotator v1.8.2 and were visualized using Fig Tree (v1.4.2) (http://tree.bio.ed.ac.uk/software/figtree/).

### Reassortment analysis

Reassortment events were analyzed using the graph incompatibility-based reassortment finder (GiRaF) program^16^. Aligned nucleotide sequence sets of the eight genetic segments were used for the phylogenetic relationship reconstruction in MrBayes (GTR+I+Γ, 1,000,000 iterations, 10% burn-in and sampling every 10,000 iterations)^17^,^18^. The trees were then used for the phylogenetic uncertainty modeling using the GiRaF program. It was repeated 10 times, and the results were summarized using the catalog reassortment events exhibiting more than 0.6 detection frequency.

### Plasmids and viruses

Two vaccine viruses, B/Brisbane/60/2008 (Victoria lineage, Bris-Vc) and B/Wisconsin/01/2010 (Yamagata lineage, Wisc-Ym), were obtained from Korea Centers for Disease Control and Prevention (Osong, Republic of Korea). After propagation in embryonated chicken eggs and purification by a plaque assay in MDCK cells (ATCC, Manassas, VA, USA), viral RNA sequences of the eight gene segments of these viruses were determined and used for the full gene plasmid construction into a bidirectional pDZ plasmid^19^. Using these plasmids, we generated PB1-, PB2-, and/or HA-reassorted viruses on either Bris-Vc or Wisc-Ym genetic backbone by reverse genetics^20^ and confirmed the correct genetic replacement of the target gene(s) from one lineage to the other using a reverse transcription PCR method. The rW/B:PB1,PB2,HA virus was not generated from three independent rescue trials.

### Mini-replicon assay

To investigate the polymerase activity of viral RNP complexes (PB1, PB2, PA, and NP genes), we additionally constructed a Gaussia luciferase reporter plasmid (pPolI-GLuc)^21^. The RNP complex gene and the pPolI-GLuc plasmids were transfected into 5 × 10^4^ 293T cells (ATCC, Manassas, VA, USA) using X-tremeGENE (Roche, Basel, Switzerland), and the cells were incubated at 37 °C for 24 h. After treating the cells using BioLux Gaussia Luciferase Assay Kit (New England Biolabs, Ipswich, MA, USA), luciferase activity was measured on a SpectraMax L plate reader (Molecular Devices, Sunnyvale, CA, USA). The cells transfected with empty plasmids were used as background luciferase expression. Luciferase activity was normalized by WST-1 treatment (Roche, Basel, Switzerland)^22^.

### Growth kinetics of the viruses in cells

To compare the replication capacity of reassorted viruses, we analyzed the growth kinetics of the viruses in MDCK cells^20^. Briefly, MDCK cells were infected with each virus at a multiplicity of infection of 0.001. After 1 h, the cells were washed five times and maintained with viral growth media at 37 °C for 48 h. Cell supernatants were then collected at 8, 16, 24, and 48 hpi and used for the titration of viral replication in MDCK cells by the plaque assay.

## Additional Information

**How to cite this article**: Kim, J. I. *et al.* Reassortment compatibility between PB1, PB2, and HA genes of the two influenza B virus lineages in mammalian cells. *Sci. Rep.* **6**, 27480; doi: 10.1038/srep27480 (2016).

## Supplementary Material

Supplementary Information

Supplementary Data S1

## Figures and Tables

**Figure 1:**
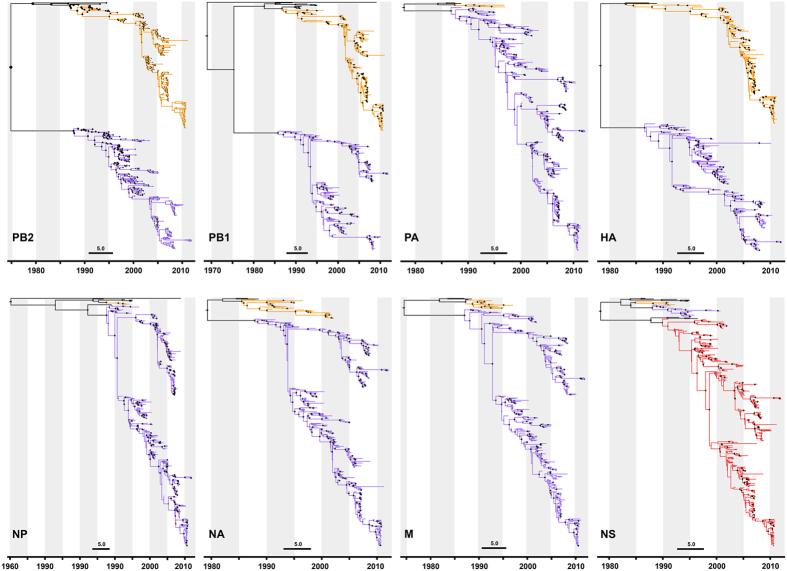
Phylogenetic relationships of the eight genetic segments of IBVs. Phylogenetic relationships of the eight genetic segments of IBVs (n = 431) were reconstructed using a time-resolved Bayesian evolution method. Colors represent different evolutionary lineages (Black, early strain circulating before Victoria and Yamagata lineage bifurcation; light orange, Victoria lineage; light purple, Yamagata lineage; and light red, Czechoslovakia lineage).

**Figure 2:**
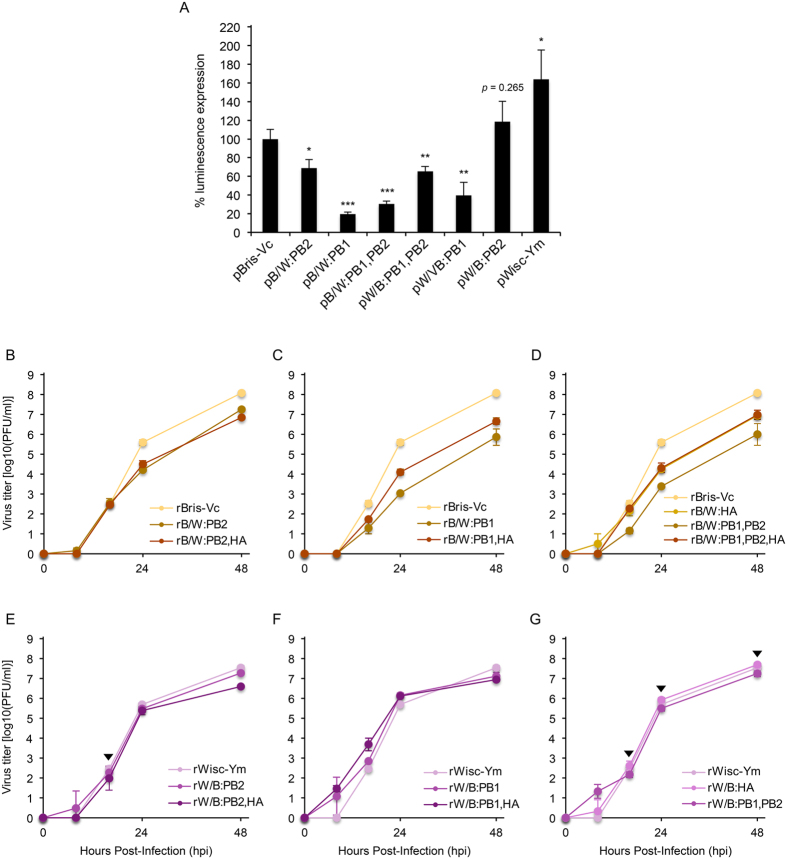
Polymerase activity and replication property of PB1, PB2, and/or HA reassortants of IBVs. (**A**) Relative activity of combinations between B/Brisbane/60/2008 (B, Victoria lineage) and B/Wisconsin/01/2010 (W, Yamagata lineage) RNP complex genes (PB1, PB2, PA, and NP) was determined in 293T cells based on the luminescence expression of the Bris-Vc RNP complex. The name in the x-axis represents a combination of RNP plasmids (abbreviated as ‘p’) (e.g., pB/W:PB1 represents a combination of Bris-Vc RNP plasmids harboring the Wisc-Ym PB1 instead of the Bris-Vc PB1). (**B–G**) Replication kinetics of PB1-, PB2-, and/or HA-reassorted viruses on the genetic backbone of Bris-Vc or Wisc-Ym virus was determined in MDCK cells. The results were represented from the mean values of three independent experiments. The name of a virus (abbreviated as ‘r’) in the x-axis is represented with the same annotation of the RNP complex combinations explained above. Statistical significance of the results (for polymerase activity, against the wild-type Bris-Vc RNP complex and for replication kinetics, against either rBris-Vc or rWisc-Ym virus) was assessed using a Student’s *t* test (*p < 0.05; **p < 0.01; and ***p < 0.001). For the growth kinetics analysis, most results exhibited statistical significance (at least, *p* < 0.05) except for the viruses indicated with an arrowhead (*p* values of rW/B:PB2, 0.32 at 16 hpi; rW/B:PB2,HA, 0.26 at 16 hpi; rW/B:HA, 0.26 and 0.14 at 16 and 48 hpi, respectively; and rW/B:PB1,PB2, 0.09 and 0.08 at 24 and 48 hpi, respectively). Error bars denote standard deviation.

**Table 1:** Reassortment events detected between the genomic segments of Victoria and Yamagata lineage viruses.

Years	Candidate virus	Gene constellation§								Reassortment event between segments		% ratio
		PB2	PB1	PA	HA	NP	NA	M	NS	Virus 1	Virus 2	
1992–1995	B/Oita/15/1992	V	E	Y	Y	E	V	V	E	M	NS, PB1, PB2	100
	B/New York/24/1993	Y	V	Y	Y	Y	V	Y	C	HA, NP	NA, PA, PB1, PB2	11.11
	B/Johannesburg/06/1994	Y	V	Y	Y	Y	V	Y	C	HA, M, NP	NA, PB1, PB2	11.11
										HA, M, NP	NA, PA, PB1, PB2	77.78
	B/Oita/15/1992	V	E	Y	Y	E	V	V	E	HA, NA, PA	PB1, PB2	12.5
	B/Ann Arbor/1994	V	E	Y	Y	E	V	V	E	HA, M, NA, PA	PB1, PB2	62.5
	B/Lisbon/02/1994	V	E	Y	Y	E	V	V	E	HA, M	PB1, PB2	12.5
	B/Connecticut/02/1995	V	E	Y	Y	E	V	V	E	HA, M, NA	PB1, PB2	12.5
1998–2005	B/Beijing/76/98	Y	Y	Y	Y	Y	Y	Y	Y	HA, M, NA, PB1, PB2	PA	30
	B/Hong Kong/542/2000	Y	Y	Y	Y	Y	Y	Y	Y	HA, NA, PB1, PB2	PA	60
	B/Hong Kong/557/2000	Y	Y	Y	Y	Y	Y	Y	Y	HA, M, NA, NS, PB1, PB2	PA	10
	B/Johannesburg/69/2001	Y	Y	Y	Y	Y	Y	Y	C			
	B/Argentina/132/2001	Y	Y	Y	Y	Y	Y	Y	C			
	B/Moscow/16/2002	Y	Y	Y	Y	Y	Y	Y	C			
	B/Ulan-Ude/6/2003	Y	Y	Y	Y	Y	Y	Y	C			
	B/Taiwan/1484/2001	V	V	Y	V	Y	V	Y	C	HA	NA, NS, PA	100
	B/Nebraska/1/01	Y	Y	Y	Y	Y	Y	Y	C	HA, PB1, PB2	NP	88.89
										HA, NS, PB1, PB2	NP	11.11
	B/Johannesburg/69/2001	Y	Y	Y	Y	Y	Y	Y	C	HA, NS	M, NA, PA, PB1, PB2	12.5
	B/Argentina/132/2001	Y	Y	Y	Y	Y	Y	Y	C	HA, NP, NS	M, NA, PA, PB1, PB2	87.5
	B/Moscow/16/2002	Y	Y	Y	Y	Y	Y	Y	C			
	B/Ulan-Ude/6/2003	Y	Y	Y	Y	Y	Y	Y	C			
	B/Houston/B69/2002	Y	Y	Y	Y	Y	Y	Y	C	HA, NA, PA	M, NP, NS, PB1, PB2	100
	B/Chile/3162/2002	Y	Y	Y	Y	Y	Y	Y	C			
	B/Malaysia/24296/2003	Y	Y	Y	Y	Y	Y	Y	C	HA, NA, NP	M, NS, PA, PB1, PB2	33.33
	B/Malaysia/24651/2003	Y	Y	Y	Y	Y	Y	Y	C	HA, NA, NP	M, PA, PB1, PB2	66.67
	B/Malaysia/24157/2003	Y	Y	Y	Y	Y	Y	Y	C			
	B/Malaysia/24666/2004	Y	Y	Y	Y	Y	Y	Y	C			
	B/Chantaburi/218/2003	Y	Y	Y	Y	Y	Y	Y	C	HA	M, NA, PB1, PB2	100
	B/Jilin/20/2003	Y	Y	Y	Y	Y	Y	Y	C			
	B/Jiangsu/10/2003	Y	Y	Y	Y	Y	Y	Y	C			
	B/Taiwan/72068/2004	Y	Y	Y	Y	Y	Y	Y	C			
	B/Taiwan/3395/2004	Y	Y	Y	Y	Y	Y	Y	C			
	B/Taiwan/125/2004	Y	Y	Y	Y	Y	Y	Y	C			
	B/Taiwan/539/2005	Y	Y	Y	Y	Y	Y	Y	C			
2006–2011	B/South Carolina/NHRC0001/2006	V	V	Y	V	Y	Y	Y	C	M, NA, NP, NS	PA	100
	B/California/NHRC0001/2006	V	V	Y	V	Y	Y	Y	C			
	B/Texas/NHRC0001/2006	V	V	Y	V	Y	Y	Y	C			
	B/Missouri/NHRC0001/2006	V	V	Y	V	Y	Y	Y	C			
	B/Mississippi/UR06-0348/2007	V	V	Y	V	Y	Y	Y	C			
	B/Illinois/UR06-0016/2007	V	V	Y	V	Y	Y	Y	C			
	B/Mississippi/UR06-0345/2007	V	V	Y	V	Y	Y	Y	C			
	B/Mississippi/UR06-0317/2007	V	V	Y	V	Y	Y	Y	C			
	B/Texas/UR06-0541/2007	V	V	Y	V	Y	Y	Y	C			
	B/Mississippi/UR06-0551/2007	V	V	Y	V	Y	Y	Y	C	HA, NA, NS, PA	M, NP, PB1, PB2	100
	B/Tennessee/UR06-0407/2007	V	V	Y	V	Y	Y	Y	C	HA, M, NA, NP, NS, PB1, PB2	PA	100
	B/Guangzhou/01/2007	Y	Y	Y	Y	Y	Y	Y	C	HA, M, NA, NP, PA, PB1, PB2	NS	100
	B/Mississippi/UR06-0477/2007	V	V	Y	V	Y	Y	Y	C	HA, PA, PB1, PB2	M, NA, NP, NS	100
	B/Mississippi/UR06-0551/2007	V	V	Y	V	Y	Y	Y	C	HA, PA	M, NP	100
	B/Mississippi/UR06-0477/2007	V	V	Y	V	Y	Y	Y	C			
	B/Mississippi/UR06-0551/2007	V	V	Y	V	Y	Y	Y	C	HA, PA	PB1, PB2	100
	B/Boston/DB02/2011	V	V	Y	V	Y	Y	Y	C			
	B/Boston/DB02/2011	V	V	Y	V	Y	Y	Y	C	HA, M, NP, PA	NA, NS, PB1, PB2	100

^§^Genetic lineages of each gene segment are represented by one letter abbreviation: E, early strain genes before Victoria and Yamagata lineage bifurcation; V, Victoria lineage genes; and Y, Yamagata lineage genes.
