# Supplementary material for: Reassortment compatibility between PB1, PB2, and HA genes of the two influenza B virus lineages in mammalian cells
Source: Sci Rep. 2016 Jun 8;6:27480. doi: 10.1038/srep27480 (PMC4897687; doi:10.1038/srep27480)
Supplement: Supplementary Information [file srep27480-s1.pdf]

Supplementary information

Reassortment compatibility between PB1, PB2, and HA genes of the two influenza B virus lineages in mammalian cells

Authors: Jin Il Kim,[1†] Ilseob Lee,[1†] Sehee Park,[1] Joon-Yong Bae,[1] Kirim Yoo,[1] Philippe Lemey,[2] Mee Sook Park,[1] Jin-Won Song,[1] Sun-Ho Kee,[1] Ki-Joon Song,[1] and Man-Seong Park[*,1]

Figure S1 Page 2
Figure S2 Page 4

Supplementary figures

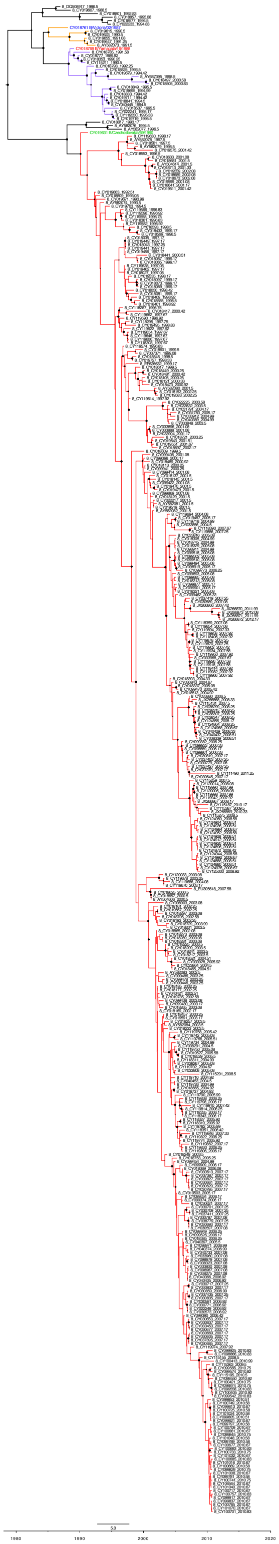

fig. S1. Phylogenetic relationship of the NS gene segment of IBVs. Phylogenetic relationship of the NS gene segment of IBVs (n = 431) were reconstructed using a time-resolved Bayesian evolution method. Colors represent different evolutionary lineages (Black, early strain circulating before Victoria and Yamagata lineage bifurcation; light orange, Victoria lineage; light purple, Yamagata lineage; and light red, Czechoslovakia lineage). A GenBank accession number was indicated for each gene sequence.

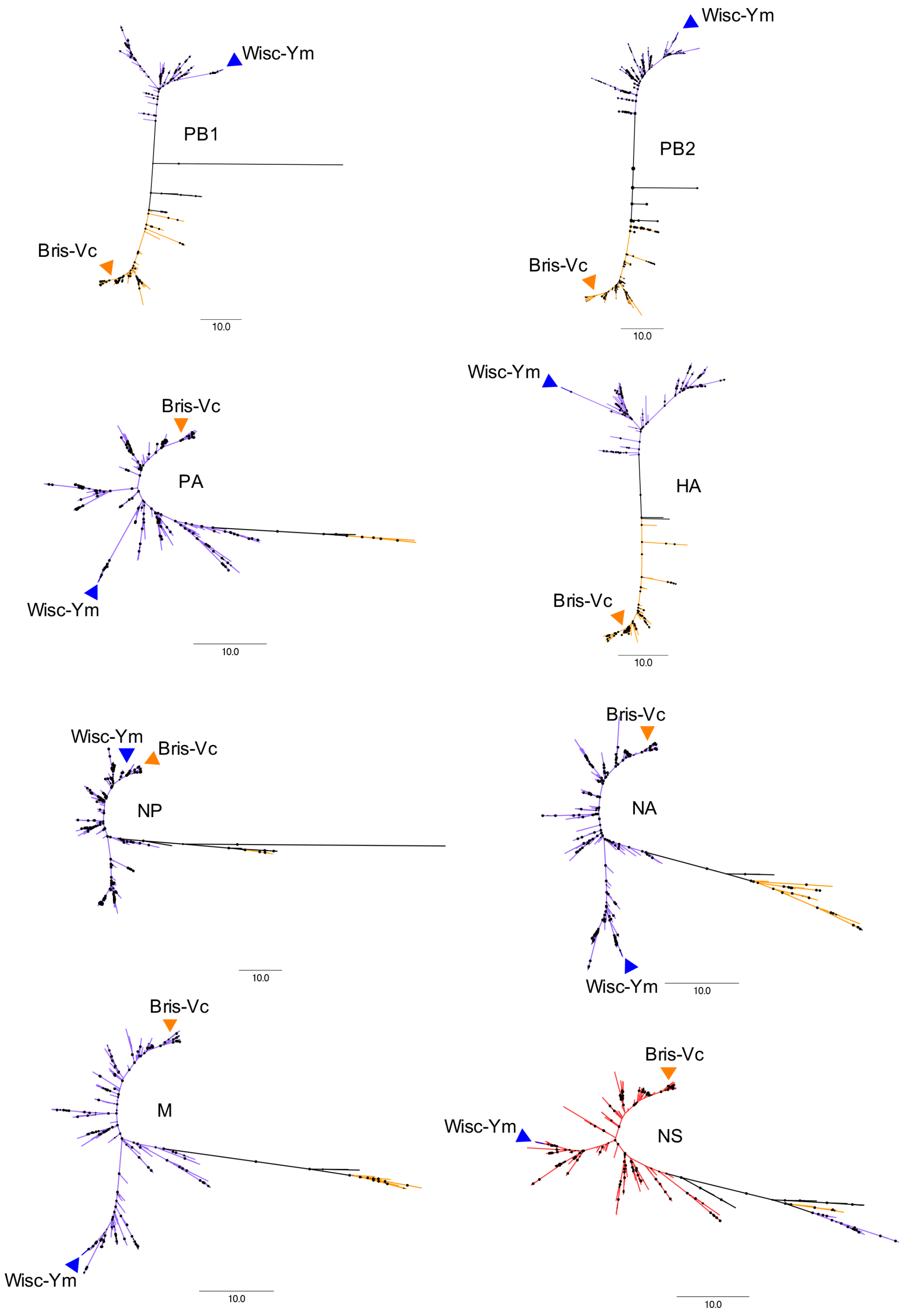
Wisc-Ym
PB1
Bris-Vc
10.0
Wisc-Ym
PB2
Bris-Vc
10.0
Bris-Vc
PA
Wisc-Ym
10.0
Wisc-Ym
HA
Bris-Vc
10.0
Wisc-Ym
Bris-Vc
NP
10.0
Bris-Vc
NA
Wisc-Ym
10.0
Bris-Vc
M
Wisc-Ym
10.0
Bris-Vc
Wisc-Ym
NS
10.0

fig. S2. Phylogenetic placements of each genetic segment of two IBV vaccine viruses. Phylogenetic locations of each genetic segment of two vaccine viruses (Victoria lineage B/Brisbane/60/2008, Bris-Vc and Yamagata lineage B/Wisconsin/01/2010, Wisc-Ym) that were used for polymerase activity and growth kinetics analyses were represented using the same MCC trees of Fig. 1.
